# Developing and testing high-efficacy patient subgroups within a clinical trial using risk scores

**DOI:** 10.1002/sim.8665

**Published:** 2020-07-14

**Authors:** Svetlana Cherlin, James M.S. Wason

**Affiliations:** 1 Newcastle Clinical Trials Unit, Newcastle University, Newcastle upon Tyne, UK; 2 Population Health Sciences Institute, Newcastle University, Newcastle upon Tyne, UK; 3 MRC Biostatistics Unit, Cambridge Institute of Public Health, Cambridge, UK

**Keywords:** adaptive design, clinical trials, risk scores, subgroup analysis

## Abstract

There is the potential for high-dimensional information about patients collected in clinical trials (such as genomic, imaging, and data from wearable technologies) to be informative for the efficacy of a new treatment in situations where only a subset of patients benefits from the treatment. The adaptive signature design (ASD) method has been proposed for developing and testing the efficacy of a treatment in a high-efficacy patient group (the sensitive group) using genetic data. The method requires selection of three tuning parameters which may be highly computationally expensive. We propose a variation to the ASD method, the cross-validated risk scores (CVRS) design method, that does not require selection of any tuning parameters. The method is based on computing a risk score for each patient and dividing them into clusters using a nonparametric clustering procedure.We assess the properties of CVRS against the originally proposed cross-validated ASD using simulation data and a real psychiatry trial. CVRS, as assessed for various sample sizes and response rates, has a substantial reduction in the computational time required. In many simulation scenarios, there is a substantial improvement in the ability to correctly identify the sensitive group and the power of the design to detect a treatment effect in the sensitive group.We illustrate the application of the CVRS method on the psychiatry trial.

## 1 Introduction

It is increasingly common in clinical trials to collect a large amount of potentially high-dimensional data about patients such as genomic, imaging, and data from wearable technologies. There is the potential for this information to be informative for explaining heterogeneity in the effect of a new treatment against control. For example, genetic signatures that are constructed based on a combination of multiple variables such as gene expression profiling, have been used to determine a subpopulation in which the novel treatment is efficacious.^1–4^

The adaptive signature design (ASD)^2^ allows a trial to develop and test efficacy of a treatment in a high-efficacy group of patients (the sensitive group) using two stages: the first stage is used to build a genetic signature, and in the second stage, the signature is applied to select the sensitive group. An extension to the ASD embeds the signature development into a cross-validation procedure.^3^ This cross-validated ASD method (CVASD) allows a more efficient use of data by increasing the sample size for developing a signature, in comparison to the original two-stage ASD method. However, the development of the genetic signature requires selection of three tuning parameters which is incorporated into each loop of the cross-validation, using a nested cross-validation procedure which may be time-consuming in practice. In addition, the approach applies the same threshold for inclusion of the genes, therefore the same genes are included into the signature for all the patients. This has the result that the signature does not necessarily efficiently use all the information from the genetic profile. For example, it has been shown that genetic signatures with good predictive abilities are not unique and may even comprise nonoverlapping genes.^5^ Therefore, incorporating flexibility in the selection of the variables has the potential to improve the predictive ability of the signature.

Motivated bythe CVASD,we propose a method for identifying the sensitive group in the trialswhere high-dimensional covariate data (such as gene expression data) are available. Similarly to the CVASD, it can be applied to a variety of types of high-dimensional data, not necessarily genetic, as we illustrate later. The method is based on a polygenic risk scoring approach that has been widely used in genetics to summarize a genetic signal among a collection of single nucleotide polymorphism markers that do not individually achieve significance.^6–13^ In the method, polygenic risk scores (RS) are constructed by computing the sum of associated alleles within each patient weighted by their effects while the effects are estimated using a different sample.^14^ Polygenic RS have previously been used for prediction of treatment response,^15,16^ prediction of disease risk^17,18^ and detection of pleiotropy in human complex traits.^6,19,20^ In the context of genetic data in cancer clinical trials, a similar method known as a compound covariate predictor, has been used for prediction of predefined tumor classes using gene expression profiles frommicroarray experiments. In thismethod, the compound covariate was constructed using test statistic for genes that are differentially expressed between the tumor classes.^1^ Compound covariate scores have also been used for quantitatively estimating treatment effects and for predicting survival curves using Cox proportional hazard model.^4,21^

In this work, we use RS to qualitatively classify patients as sensitive or not using a nonparametric clustering approach. We apply the concept of polygenic RS to high-dimensional covariate data and construct the RS as sums of the covariates weighted by their estimated effects. Our method, hereafter called cross-validated risk scores (CVRS), employs a cross-validation procedure. Within each one of the cross-validation folds, RS are constructed and partitioned into two groups using the *k*-means clustering procedure.^22^ *K*-means is a heuristic procedure that starts with randomly selected initial cluster centers and iteratively optimizes the within-cluster sum of squares, where the optimization process is built-in within the *kmeans* R function, and therefore no parameter tuning is required when applying the function.

For a large number of covariates, a preselection might be beneficial. This is commonly done using a P-value threshold,^19^ however, there is no general agreement about the best threshold. For example, while *P* <.5 has been used to construct polygenic RS for distinguishing schizo-affective cases from other bipolar disorder cases,^8^ all known inflammatory bowel disease associations (*P* < 1) have been used to distinguish between cases with colonic Crohn’s disease, ileal Crohn’s disease, and ulcerative colitis.^11^ The threshold of *P* <.01 has been used for constructing a genetic signature to predict survival after chemotherapy for diffuse large-B-cell lymphoma,^23^ while the threshold of *P* <.001 has been used in the context of predicting survival of patients with metastatic kidney cancer^24^ and survival of patients with multiple myeloma.^4^ Different P-value thresholds have been investigated to predict the genetic risk for a variety of diseases (bipolar disorder, coronary heart disease, hypertension, type I and II diabetes, rheumatoid arthritis), and it has been found that the *P*-value threshold is disease-specific.^7^ Another approach for gene filtering is incorporating variable selection algorithm (such as lasso^25^) into the estimation of parameters.^26^ However, it has been shown that univariate gene selection approaches often achieve better results than multivariate approaches.^27,28^ In this study, we investigate the benefits of adding an additional selection step using a *P*-value threshold method applied to real data from a psychiatry clinical trial.

We have developed an open-source R package *rapids* (Risk-score AdaPtIve DeSign) that implements the existing CVASD method^3^ and our new CVRS method (to the best of our knowledge, there is currently no implementation of the existing CVASD method). The methods are presented in terms of a binary outcome, but can be adapted to other types of outcomes (eg, normally distributed or time-to-event end points).

The remainder of this article is organized as follows. The Methods section describes the existing CVASD design and the newCVRS design. In the SimulationResults section,we explore the operating characteristics of the two designs for various simulation scenarios. In the Real Data Example section, we illustrate the application of the designs to a psychiatry clinical trial that shows that the method can be broadly applicable beyond genetic data. Finally, we summarize our conclusions in the Discussion section.

## 2 Methods

We employ the modeling assumptions of the existing ASD design^3^ whereby for a binary outcome, the response to treatment is influenced by a subset of *K* unknown covariates (the sensitive covariates) through the following model:

$$\log\left(\frac{p_{i}}{1-p_{i}}\right)=\mu +\lambda t_{i}+\alpha_{1}x_{i1}+\ldots +\alpha_{K}x_{iK}+\gamma_{1}t_{i}x_{i1}+\ldots +\gamma_{K}t_{i}x_{iK},$$

where *p_i_* is the probability of response to treatment for the ith patient; *μ* is the intercept; *λ* is the treatment main effect that all patients experience regardless of the values of the covariates; *t_i_* is the treatment that the ith patient receives (*t_i_* = 0 for the control arm and *t_i_*=1 for the treatment arm); *x*_*i*1_, …, *x_iK_* are the values for the *K* unknown sensitive covariates; *α*_1_, …, *α_K_* are main covariate effects for the *K* covariates; *γ*_1_, …, *γ_K_* are treatment-covariate interaction effects for the *K* covariates. The model assumes that there is a subset of patients (the sensitive group) with a higher probability of response when treated with the new treatment compared with the control treatment.

### 2.1 CVASD design

In the CVASD design, a 10-fold cross-validation procedure is performed to develop a signature with which to classify patients as either sensitive or not. In each fold of the cross-validation procedure, 9/10 of the data (training subset) are used to develop a signature, with the other 1/10 of the data (testing subset) held out to be used for applying the signature to identify a sensitive group. The procedure is then repeated 10 times with a different 1/10 of the data being held out, such that at the end of the cross-validation procedure each patient is classified as either sensitive or not. For each covariate *j* in the training subset, a single-covariate logistic regression model is fit:

$$\log\left(\frac{p_{i}}{1-p_{i}}\right)=\mu +\lambda_{j}t_{i}+\beta_{j}t_{i}x_{ij}.$$

A covariate that has treatment-covariate interaction term *β_j_* that is significant at a prespecified level *η* is classified as sensitive. The predicted new vs control arm odds ratio $\exp\left({\hat{\lambda }}_{j}+{\hat{\beta }}_{j}x_{j}\right)$ is computed for each one of the sensitive covariates in the testing subset, where ${\hat{\lambda }}_{j}$ and ${\hat{\beta }}_{j}$ are estimated treatment effect and treatment-covariate interaction effect, respectively. A patient is designated sensitive if the odds ratio $\exp\left({\hat{\lambda }}_{j}+{\hat{\beta }}_{j}x_{j}\right)$ exceeds a prespecified threshold *R* for at least *G* of the sensitive covariates. The development of the signature requires specification of a set of the three previously defined tuning parameters: *η*, *R,* and *G* (the tuning set). Selection of the tuning set is incorporated into each loop of the cross-validation using a nested cross-validation procedure as follows. First, a list of possible tuning sets is prespecified. For each training subset, an inner (nested) loop of the cross-validation procedure is applied to obtain a sensitive group of patients, corresponding to each tuning set. A tuning set corresponding to the sensitive group with the smallest *P*-value (for the difference between arms) is then selected for the use in the training subset of the main cross-validation procedure. A drawback of the nested cross-validation procedure is its computational intensity. The computational time of the procedure increases as the number of the plausible tuning sets increases. In the absence of the prior knowledge about the values of the tuning set, the grid of values considered in the list of the tuning sets needs to be large to adequately cover the space of the potential values of the parameters. As a result, a large number of plausible tuning sets needs to be evaluated, which might require a long computational time. To save computational time, it has been proposed^3^ to use only one outer cross-validation training subset to select the tuning set. In our implementation of the CVASD method, we use only one inner cross-validation fold within each outer cross-validation fold instead. This simplification reduces the computational time similarly to the original simplification, while also preserving the validity of the entire nested cross-validation procedure.

For the simulated data, we test for a difference between the arms using a test based on a normal approximation for the difference of two proportions for the overall level test (*prop.test* R function, two-sided), carried out at a significance level *α*_1_, and Fisher’s exact test for the subgroup level test (*fisher.test* R function, two-sided) carried out at a significance level *α*_2_. The overall procedure is considered positive if either of these tests is significant, and the overall significance level is *α* = *α*_1_ + *α*_2_. In our study we use *α*_1_ = 80% of *α* and *α*_2_ = 20% of *α*, as recommended for the ASD design.^2^ However, our implementation allows for different allocations of *α*_1_ and *α*_2_. For the real data, we test for the interaction effect between the treatment and the sensitivity status, using a generalized linear model with a binomial link function (*glm* R function) and the same partitioning of *α.*

Because the sensitive group was obtained by cross-validation, we use the permutation method^29^ to obtain a valid *P*-value for testing the interaction effect between the treatment and the sensitivity status in the analysis of the real data. The permutation *P*-value is given by

(1) $$\frac{1+\text{number}\;\text{of}\;\text{elements}\;\text{of}P*\leq P_{0}}{1+\text{number}\;\text{of}\;\text{permutations}},$$

where P* is the vector of the *P*-values for the treatment-sensitivity status interaction effect computed for 2000 permuted datasets, and *P*_0_ is the *P*-value for the treatment-sensitivity status interaction effect obtained for the original (nonpermuted) data. Here, each permutation dataset was obtained by randomly permuting the treatment labels of the original data.

### 2.2 CVRS design

The proposed CVRS design develops a signature based on the construction of RS. The design consists of two steps. First, the RS are constructed as sums of associated covariates within each patient weighted by their estimated effects. In the second step, a *k*-means clustering procedure is applied to divide the RS into two clusters that correspond to sensitive and nonsensitive groups of patients. For constructing the RS, we employ the cross-validation procedure, in which we build the model using the training subset and use this model to compute the RS for the testing subset as follows. For *r*-fold cross-validation, the observed dataset *D* is randomly divided into *r* nonoverlapping subset *D* ^(*l*)^ *l* = 1, …, *r*, of (approximately) equal size *N/r.* For each iteration of the *r*-fold cross-validation, data are split into test *D* ^(*l*)^ and training *D* ^(−*l*)^ (formed by removing *D* ^(*l*)^ from D) subsets and the coefficients for treatment by covariate interaction ${\hat{\beta }}_{j}^{\left(-l\right)}$ are estimated for each covariate *j* from Equation (2) using training data alone.

(2) $$\log\left(\frac{p_{i}}{1-p_{i}}\right)=\mu +\lambda t_{i}+\alpha_{j}x_{ij}+\beta_{j}t_{i}x_{ij}.$$

Then, for each test set *D* ^(*l*)^, the RS are computed as ${\text{RS}}_{i}^{\left(l\right)}=\Sigma_{j}{\hat{\beta }}_{j}^{\left(-l\right)}x_{ij}^{\left(l\right)}$, where $x_{ij}^{\left(l\right)}$ is the value of the covariate *j* for the ith patient in the *l*th test set. Within each test set *D* ^(*l*)^, the *k*-means procedure with *k* = 2 is applied to classify the test scores ${\text{RS}}_{i}^{\left(l\right)}$, *i* = 1, …, *N/r* into sensitive and nonsensitive groups. Therefore, at the end of the cross-validation process each patient in the observed data *D* is classified either as sensitive or nonsensitive, after pooling group membership status across the *r* test sets.

The *k*-means clustering partitions the observations into *k* clusters by assigning each observation to a cluster based on a distance between the observation and a cluster mean. The algorithm works in an iterative manner, in which the observations are moved from one cluster to another and the sum of the squares from the observations to the assigned cluster means is recomputed for every iteration. The algorithm proceeds until no reassignment of observations minimizes the within-cluster sum of squares.^30^ Here, we take *k* = 2 in order to partition the RS into two groups: sensitive and nonsensitive. However, this approach could potentially be generalized into more clusters.

For analyzing the simulated data, we did not include the main treatment and covariates effects into the model, to match the data generating mechanism, that is:

$$\log\left(\frac{p_{i}}{1-p_{i}}\right)=\mu +\beta_{j}t_{i}x_{ij}.$$

The RS are then computed as described previously $\left({\text{RS}}_{i}^{\left(l\right)}=\sum_{j}{\hat{\beta }}_{j}^{\left(-l\right)}x_{ij}^{\left(l\right)}\right)$. The tests for the treatment effect in the sensitive group, as well as the permutation procedure for obtaining valid *P*-values, are then performed similarly to that for the CVASD method.

In the analysis of the real data,we investigated the benefits of an additional *P*-value filtering step by filtering the covariates based on a *P*-value for the treatment-covariate interaction effect obtained from a covariate-wise logistic regression. To this end, we run the analysis for different *P*-value thresholds ***P*** ∈ {0.01*n*} *n* = 1, …, 100 for the treatment-covariate interaction. For each value in ***P***, we found the sensitive group and computed the *P*-value for the interaction between the treatment and the sensitivity status. Denote by ***P***_int_ a vector of the *P*-values for testing the interaction between the treatment and the sensitivity status that corresponds to the values of ***P***, and denote by ${\hat{P}}_{0}$ the value of ***P*** that minimizes ***P***_int_, that is

$${\hat{P}}_{0}=\underset{P}{\arg\min}\;P_{\text{int}}.$$

Then, $P_{\mathrm{int}}\left({\hat{P}}_{0}\right)$ corresponds to the minimum *P*-value for the treatment-sensitivity status interaction effect computed using the threshold ${\hat{P}}_{0}$ for filtering the covariates.

To account for the additional *P*-value filtering step in the permutation analysis, the permutation *P*-value for the interaction effect between the treatment and the sensitivity status was obtained as follows. For every permuted dataset *d*, we performed the entire CVRS design procedure for different *P*-value thresholds ***P*** and we obtained $P_{\mathrm{int}}^{\left(d\right)}\left({\hat{P}}^{\left(d\right)}\right)$ that corresponds to the minimum *P*-value for the treatment-sensitivity status interaction effect computed using the threshold ${\hat{P}}^{\left(d\right)}$ for filtering the covariates. Denote by $P_{\mathrm{int}}^{*}=\left\{{\hat{P}}_{\mathrm{int}}^{\left(d\right)}\left({\hat{P}}^{\left(d\right)}\right)\right\}$ a vector of $P_{\mathrm{int}}^{\left(d\right)}\left({\hat{P}}^{\left(d\right)}\right)$ for *d* = 1, …, 2000. The permutation *P*-value is given by

(3) $$\frac{1+\text{number}\;\text{of}\;\text{elements}\;\text{of}P_{\mathrm{int}}^{*}\leq {\hat{P}}_{0}}{1+\text{number}\;\text{of}\;\text{permutations}}.$$

## 3 Simulation Results

We conducted a simulation study to evaluate the performance of the CVASD and the CVRS, using 10-fold cross-validation. We assumed a clinical trial with 100 independent covariates where *K*=10 of them are sensitive (eg, this can represent a 100-genes array with 10 sensitive genes). The reason for the assumption of independence was motivated by the previous studies^2,3^ that achieved similar results with correlated data. The main effects of the covariates were assumed to be 0, and the treatment-covariate interaction effects were assumed to be constant across the sensitive covariates (*γ*_1_ = *γ*_2_ = … = *γ_K_*), similarly to the CVASD.^3^ An intercept *μ* was set to correspond to a control arm response rate of 25%. We used an overall significance level *α* = 0.05 (two-sided) that corresponds to *α*_1_ = 0.04 and *α*_2_ = 0.01 significance levels for the overall test and for the sensitive group test, respectively. The empirical power of the adaptive designs was calculated as the percentage of replications with either a positive overall 0.04 level test or a positive 0.01 level sensitive group test. The simulations were based on 1000 replications (see Appendix A for details on how data were simulated).

First, we considered a situation where the sensitive group consisted of 10% of patients, and only the sensitive group benefited from the new drug. Table 1 presents the results corresponding to the 70%, 60%, and 50% response rates in the sensitive group under treatment. As expected, the higher the response rate in the sensitive group under treatment, the higher the power of both methods. Yet, theCVRS systematically outperformed theCVASD in identification of the sensitive group, as measured by the sensitivity and specificity of the group selection. As a result, the response rate in the sensitive group under treatment was more precisely estimated with the CVRS than with the CVASD. In addition, the power for the sensitive group and the overall power of the adaptive design were higher with the CVRS for many scenarios.We also tried higher response rates (80% and 98%) and found that, despite both designs achieving good operating characteristics, the CVRS outperformed the CVASD. For example, for 98% response rate and sample size 1000, both designs had a power of 1 for sensitive group level test. However, while for 80% response rate the CVRS had a power of 1, the power of the CVASD dropped to 0.839.

We next considered a scenario where nonsensitive patients show a slight benefit on the treatment arm (35% response rate), and the sensitive group is larger than in the previous scenario (20%; Table 2). For both 50% and 60% response rates in the sensitive group under treatment, the overall 0.04 level test achieved high power with both methods, therefore the overall power of the adaptive design was also high for both methods. However, the sensitive group was substantially better identified with the CVRS. For example, for sample size 1000 with 50% response rate in the sensitive group under treatment, the sensitivity and specificity of selecting the sensitive group took the values of 0.649 and 0.653 for the CVASD, and the values of 0.98 and 0.989 for the CVRS. The good separation ability of the CVRS resulted in a large improvement in the power for the sensitive group (0.824 for the CVRS in comparison to 0.572 for the CVASD).

Interestingly, Table 3 shows that for a smaller proportion of sensitive patients (such as 5%), a better separation achieved with the CVRS might result in a small loss of power in comparison to the CVASD (eg, for sample size 1000 with 60% response rate). This is likely because the CVASD classified more nonsensitive patients in the sensitive group (false positives), as evident from the specificity of the group selection being 0.783 (the corresponding specificity for the CVRS is 0.946). Including a large number of false positives into the small sensitive group drastically inflated its size. This (counter-intuitively) increased the power for the sensitive group because all patients benefited from the treatment. However, the treatment effect in the sensitive group under treatment was diluted and was estimated less precisely in comparison to the CVRS (0.448 for the CVASD and 0.546 for the CVRS when the true response rate was 0.6).

In the situation where the response rates on both the control arm and the nonsensitive group on the treatment arm are 35% (Table 4) the power achieved with the CVRS is, in some cases, two to three times higher than the power achieved with the CVASD, due to a better identification of the sensitive group.

In the null scenario of no sensitive group, the CVRS more accurately identified no sensitive group effect as evident from the sensitivity and specificity values of 0.5, in comparison to the CVASD, where the corresponding values differed from 0.5 (Supplementary Table 1). In addition, the type I error was well controlled with the CVRS (estimated type I errors were 0.011 and 0.015 for samples sizes 400 and 1000, respectively).

**Figure 1:**
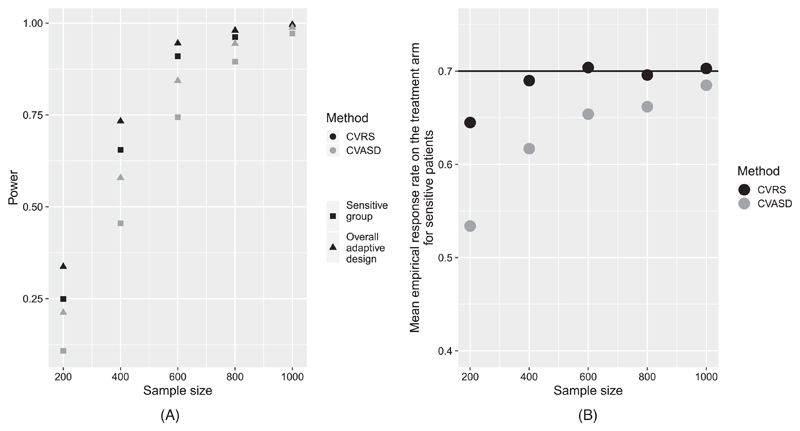
Power (A) and mean estimated response rate in the sensitive group on the treatment arm (B), for the CVRS and the CVASD for different sample sizes. In (B), the horizontal line represents the true response rate of 70%. CVASD, cross-validated ASD method; CVRS, cross-validated risk scores

We compared the power of the methods and the estimated response rate in the sensitive group under treatment, for different sample sizes (200, 400, 600, 800, and 1000). For all of the sample sizes, we investigated the scenario where the response rates in the control arm and in the nonsensitive patients under treatment are 35%, the response rate in the sensitive group under treatment is 70% and 20% of the patients are sensitive (Figure 1). While for the largest sample size (1000), the power of the two methods was comparably high, for smaller sample sizes, the power of the CVRS was systematically higher than that of the CVASD. The same trend appeared for the estimated response rate in the sensitive patients under treatment. For smaller sample sizes, the response rate in the sensitive group under treatment was more precisely estimated with the CVRS in comparison to the CVASD.

In all of the scenarios, the CVRS requires three to four times less computational time in comparison to the CVASD with three tuning sets, due to an absence of a nested cross-validation procedure (with more tuning sets, the difference in the run times of the methods will substantially increase). In addition, the mean response rate in the sensitive group on treatment is more precisely estimated with the CVRS, due to a better identification of the sensitive group.

To assess the sensitivity of the CVRS method to different data generating mechanisms, we simulated data with main treatment effect, main covariates effect (prognostic effect) and treatment-covariates interaction effects, and analyzed it by fitting models that included (i) interaction effects only; (ii) main covariates effects and interaction effects; (iii) main treatment and covariates effects and interaction effects. The results show a high performance of the model that included interaction effects only, while there is only a small reduction in power for the models that included the main effects (Supplementary Table 2). To investigate the sensitivity of the CVRS method to different levels of correlation between the covariates, we simulated data assuming a high correlation (*ρ* = 0.6) and a low correlation (*ρ* = 0.3) between the covariates. We showed that the performance of the CVRS method slightly decreases with the increased level of the correlation between the covariates (Supplementary Table 3). This makes sense as the first stage testing is one-by-one, therefore more nonassociated covariates become part of the signature when there is higher correlation. This suggests that the design might benefit from prefiltering of the covariates based on the correlation between them, which we consider more in the following section.

We note that dividing theRS into two clusters corresponds to determining the threshold for the constructed RS, thereby classifying observations into sensitive and nonsensitive based on applying *k*-means to the test data. This might in theory cause overfitting. To investigate this issue we examined nested cross-validation CVRS, where the clusters’ centers for the RS are estimated within the inner cross-validation layer as follows. For each fold in the inner cross-validation layer, the model as described by Equation (2) is fit to the training set and the RS are constructed and divided into two clusters for the test set, similarly to the cross-validation process described in Section 2.2. At the end of the inner cross-validation process, a vector of *r* clusters’ centers that correspond to *r* inner folds, is obtained for the *D* ^(−*l*)^ subset, that is, ***c_i_*** = {*c*_1,*i*_, *c*_2,*i*_}, where *i* = 1, …, *r*. For each fold in the outer cross-validation layer, the model as described by Equation (2) is fit to the training dataset *D* ^(−*l*)^ and the RS are constructed for the test dataset *D^l^* using the parameters estimated from *D* ^(−*l*)^. The RS for *D^l^* are then divided into clusters using themeans of the clusters’ centers obtained in the inner cross-validation, that is, *mean*(***c_i_***). We compared the operating characteristics of the CVRS with those of the nested CVRS for different sample sizes and found that they are very similar (Supplementary Table 4).

## 4 Real Data Example

To illustrate the application of our approach,we applied the CVRS and theCVASDmethods to the data from the systematic therapy of at risk teens (START).^31^ STARTwas a randomized controlled trial comparing the outcomes of young people and their families who were allocated to treatment as usual, hereafter referred to as control arm and multisystemic therapy, hereafter referred to as treatment arm. The trial randomized 683 participants. Theoutcomewe considered was whether or not the young person committed a criminal offence in the 18 months postrandomization. For each participant, 94 baseline covariates were collected. These consisted of a range of demographic variables, questionnaires and psychiatric diagnoses. We excluded covariates and participants with > 10% missing data. This filtering resulted in the dataset comprised of 669 participants (336 participants in the control arm and 333 participants in the treatment arm) and 86 covariates. We used mean imputation to impute remaining missing covariates. Participants with one or more offences were defined as offenders, while participants with no offenceswere defined as nonoffenders. This definition resulted in 288 offenders (143 in the control arm and 145 in the treatment arm) and 381 nonoffenders (193 in the control arm and 188 in the treatment arm), with no significant treatment effect as measured by a logistic regression (P =.797).

For the CVASD method, we transformed the values of the covariates so that higher values became always positively associated with the outcome. The transformation was based on the treatment-covariate interaction coefficient from the regression model (the covariates were multiplied by −1 if the corresponding coefficients were negative). For the CVRS method, this transformation was not needed because the method itself includes multiplying the covariates by the corresponding coefficients. The CVASD algorithm implemented with six tuning sets {(0.01, 2, 3), (0.02, 3, 2), (0.03, 4, 1), (0.5, 1.5, 2), (0.6, 1.6, 2), (0.7, 1.7,1)}, found a sensitive group comprised of 70 participants (34 participants in the control arm and 36 participants in the treatment arm), with no significant interaction effect between the treatment and the sensitivity status (permutation-based *P* =.752). The CVRS method indicated the existence of a sensitive group comprised of 453 participants (222 participants in the control arm and 231 participants in the treatment arm) with no significant interaction effect between the treatment and the sensitivity status (permutation-based *P =*.122 as obtained by Equation 1).

**Figure 2:**
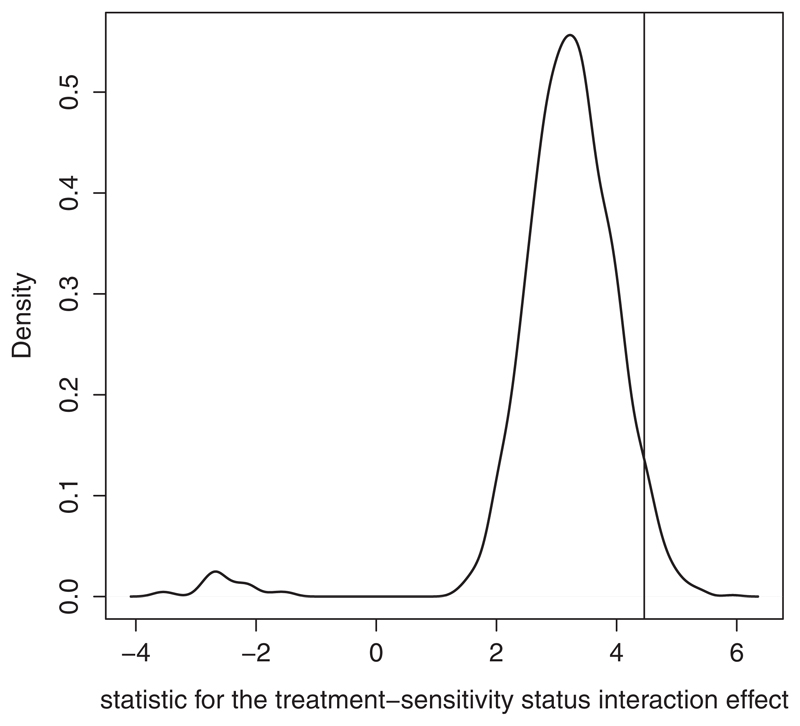
Density plot of 2000 test statistics that correspond to $P_{\mathrm{int}}^{*}$. The vertical line represents the value of the statistic for the original (nonpermuted) data that corresponds to ${\hat{P}}_{0}$ (4.46)

In the analysis that included the prefiltering of the covariates, *P*-value threshold of.14 gave the smallest *P*-value for the interaction between treatment and sensitivity status (*P* = 8.2 × 10^−6^) and it resulted in 16 covariates (see Supplementary Table 5). Using this threshold, the CVRS method found a sensitive group comprised of 584 participants (294 participants in the control arm and 290 participants in the treatment arm), with the permutation-based *P* =.043 as obtained by Equation (3). The numbers of participants in each arm who are sensitive/nonsensitive for both methods are reported in Supplementary Table 6. We note that the null distribution of the test statistic that corresponds to $P_{\mathrm{int}}^{*}$ is inflated due to the tuning of the *P*-value threshold for each permuted dataset (Figure 2).

## 5 Discussion

We have presented a modification of the CVASD method.^3^ Our proposed CVRS design is based on selection of the sensitive group of patients using RS. By contrast to the existing CVASD method that considers a prespecified number or covariates reaching a prespecified significance level and a prespecified odds ratio, the CVRS method allows all of the covariates to contribute to the construction of a signature, thus eliminating a need for the prior assumptions about the number of the true causal covariates, significance level and odds ratio. In the CVRS method, a single RS is constructed for each patient, which is a sum of the associated covariates weighted by their estimated effects. The RS are then divided into two clusters corresponding to sensitive and nonsensitive groups, using a nonparametric *k*-means approach. The CVRS is implemented in a cross-validation procedure. Since no parameters needed to be tuned, no nested cross-validation is required and the computational times reduced drastically in comparison to the nested cross-validation procedure implemented in the existing CVASD method (in comparison to the CVASD with three tuning sets, the CVRS achieved three to four times reduction in computational time).

To ensure that the cross-validation procedure is valid, we computed the coefficients for the RS from the training data, and then used them to compute the RS for the testing data. The RS were then divided into two groups for the testing data fold-wise.We note that dividing the RS into two groups can be thought of as determining the threshold for the constructed RS which is based on the testing data.However,wewould argue that the fact that the RS themselves are constructed based on the training sets, and the division of theRS is performed fold-wise, ensures the validity of the cross-validation procedure because it avoids contaminating the testing datawith the information from the training data. This can be supported by the fact that simulation studies that used regular *P*-values from Fisher’s exact test rather than permutation-based *P*-values, show no evidence of the inflation of the type I error (or at least not beyond Monte Carlo error from the limited number of replicates; see Supplementary Table 1). In addition, the operating characteristics of the nested CVRS design (where the clusters’ centers are estimated by the inner cross-validation layer), as compared with those of the CVRS design for different sample sizes, confirm the validity of the cross-validation procedure (see Supplementary Table 4). We note, that for a smaller sample size we encountered an issue with the nested CVRS design. Because in the nested CVRS design the clusters’ centers are supplied to the *kmeans* R function, it is possible that all the RS in the test fold may be closest to one of the centers. In this case the *kmeans* R function returns an “empty cluster” warning. To avoid a failure of the nested CVRS design for these cases, we therefore did not supply the clusters’ centers to the *kmeans* function but rather supplied a number of clusters (*k* = 2) which is equivalent to the nonnested CVRS design. However, the number of replicates in which this occurred is very small (1%).

We have investigated the performance of the CVRS method by applying it to the simulated data and found that for many scenarios, it shows a substantial improvement in the ability to correctly identify the sensitive group and the overall power of the design, in comparison to the existing CVASD method. We showed that the CVRS method more accurately estimates the response rate in the sensitive group on the treatment arm in a variety of simulation scenarios. This is due to a better identification of the sensitive group, as measured by the sensitivity and specificity of the group selection algorithm. In our simulations, we used relatively low response rates for the sensitive group on the treatment (50%, 60%, and 70%). With higher response rates such as 80%, 90%, and 98%, both methods achieved higher power, however, we felt that lower response rates are more realistic.

To illustrate the broader applicability of the method, we have applied it to the data from START randomized controlled trial.We showed that theCVRSmethod applied with an additional selection step, identified a sensitive group that conferred a nominally significant interaction effect (*P* <.05) between the treatment and the sensitivity status, in the case where there was no overall significant treatment effect. By contrast, the CVASD method implemented with six tuning sets, identified a sensitive group that did not confer a significant interaction effect between the treatment and the sensitivity status. We note that for the CVASD, different results might be obtained with different tuning sets, however, choosing the tuning sets is fraught with difficulty. To ensure that the candidate tuning sets included values that are close enough to optimal, we run a pilot run (10 replications) for a large number of different tuning sets. For the simulation study, we choose the sets that gave the highest values for the sensitivity and specificity of identifying the subgroup in the pilot runs. However, the same strategy cannot be applied for real data because the true sensitive group is unknown, hence prior knowledge about the behavior of the covariates is required. To account for the absence of the prior knowledge about the behavior of the covariates, we choose the tuning sets that included a wide grid of values to adequately cover the space of the potential values of the parameters. On the contrary, the CVRSmethod can be applied in an agnosticmanner that does not require prior assumptions regarding the covariates.

We have shown the benefit of preselecting the covariates based on the *P*-value threshold. When CVRS was implemented without the selection step to the START trial data, it was unable to identify a significant treatment effect in the sensitive group. However, when the selection step was added, the significance of the interaction effect between the treatment and the sensitivity status was below a nominal significance level of 0.05. This might be explained by the fact that the RS computed with the reduced number of covariates were less noisy in comparison to those computed from the full model. Reducing the number of the covariates can also have a substantial practical advantage. For example, many of the baseline covariates in START were questionnaires, then the number of questions in the questionnaires can be reduced in future trials: reducing the number of questionnaires needed for classifying future participants as sensitive is desirable. Here, our choice of the *P*-value threshold was guided by the significance of the treatment-covariate interaction effect. Further studies will investigate other approaches of choosing the *P*-value threshold, as well as other methods for filtering covariates (eg, according to the variability of their values^1,32^).

We note, that permutation analysis that included tuning *P*-value threshold resulted in an inflated test statistic for the treatment-sensitivity status interaction effect (Figure 2). To investigate this issue, we have examined a number of nonstandard permutations methods and nonstandard test statistics,^33^ however, these resulted in a similar inflation (data not shown). This suggests that the (inflated) permutation distribution of the test statistic as shown in Figure 2 corresponds to the true null distribution. We also note that this study does not claim to provide a psychological rationale for the existence of the sensitive group. However, these results suggest that sensitivity status found with the CVRS method, can be useful in predicting the outcome and might provide a starting point for further independent investigation.

Both theCVASD and the CVRS designs have some limitations. First, computational problems might occurwhile fitting the *glm* model due to a lack of convergence or a perfect separation.^34^ To avoid the perfect separation issue, the *brglm* function can be used.^35^ For both designs, small sensitive group and/or small benefit for the sensitive group relatively to the nonsensitive patients reduce the power of the designs. For the CVRS design, a potential issue might be the fact that the *k*-means algorithm is sensitive to the random starting assignments. To address this limitation,we suggest using a large number of starting assignments, specified by the *nstart* parameter of the *kmeans* function.

In this study, we have retrospectively applied the CVRS method to identify the sensitive group in psychiatry trial participants. In principle, the method can be used to prospectively identify whether a participant belongs to a sensitive group, thus facilitating adaptive randomization.

We have provided a freely available R package that implements our new CVRS method as well as the existing CVASD method (see Appendix B for details). In the package, the methods are developed for binary outcome and for two levels of sensitivity (sensitive and nonsensitive patients). Further research will focus on extending the implementation of the methods to incorporate different types of outcomes, and more levels of sensitivity of the patients. In addition, future work will considermore than one outcome (eg, efficacy and toxicity) using an extension of the clustering approach.

## Supplementary Material

Supplementary materials

## Figures and Tables

**Table 1:** Operating characteristics of the CVASD and the CVRS methods for different true response rates in sensitive group on the treatment arm (TRR) for the following scenario: 25% response rate on the control arm, 25% response rate in nonsensitive group on the treatment arm, 10% of the patients are sensitive

		Sample size 400		Sample size 1000	
TRR	Operating characteristics	CVASD	CVRS	CVASD	CVRS
70%	Power for overall 0.04 level test	0.144	0.144	0.271	0.271
	Power for sensitive group 0.01 level test	0.473	0.463	0.972	0.977
	Overall power of the design	0.549	0.54	0.98	0.983
	Sensitivity of the group selection	0.889	0.996	0.987	0.998
	Specificity of the group selection	0.994	0.97	1	1
	Response rate in the sensitive group	0.652	0.641	0.699	0.699
	Run time (min.)	210	44.4	315	76.7
	Tuning set	A	−	A	−
60%	Power for overall 0.04 level test	0.086	0.086	0.185	0.185
	Power for sensitive group 0.01 level test	0.199	0.246	0.785	0.848
	Overall power of the design	0.268	0.311	0.839	0.876
	Sensitivity of the group selection	0.82	0.983	0.91	0.999
	Specificity of the group selection	0.97	0.933	0.998	1
	Response rate in the sensitive group	0.514	0.523	0.602	0.612
	Run time (min.)	210.4	45.15	313.7	63.7
	Tuning set	B	−	B	−
50%	Power for overall 0.04 level test	0.047	0.047	0.109	0.109
	Power for sensitive group 0.01 level test	0.043	0.108	0.389	0.437
	Overall power of the design	0.088	0.15	0.464	0.498
	Sensitivity of the group selection	0.81	0.95	0.878	0.992
	Specificity of the group selection	0.849	0.853	0.991	0.986
	Response rate in the sensitive group	0.341	0.409	0.477	0.493
	Run time (min.)	216.8	45.32	308.9	77.5
	Tuning set	C	−	C	−

*Note:* Tuning sets are specified in Appendix A. Abbreviations: CVASD, cross-validated ASD method; CVRS, cross-validated risk scores.

**Table 2:** Operating characteristics of the CVASD and the CVRS methods for different true responses rates in sensitive group on the treatment arm (TRR) for the following scenario: 25% response rate on the control arm, 35% response rate in nonsensitive group on the treatment arm, 20% of the patients are sensitive

		Sample size 400		Sample size 1000	
TRR	Operating characteristics	CVASD	CVRS	CVASD	CVRS
60%	Power for overall 0.04 level test	0.857	0.857	0.999	0.999
	Power for sensitive group 0.01 level test	0.451	0.674	0.946	0.998
	Overall power of the design	0.921	0.953	1	1
	Sensitivity of the group selection	0.698	0.976	0.943	0.998
	Specificity of the group selection	0.868	0.979	0.864	1
	Response rate in the sensitive group	0.495	0.592	0.543	0.607
	Run time (min.)	209.6	57.1	305.9	90.2
	Tuning set	A	−	A	−
50%	Power for overall 0.04 level test	0.737	0.737	0.993	0.993
	Power for sensitive group 0.01 level test	0.26	0.346	0.572	0.824
	Overall power of the design	0.805	0.828	0.997	0.999
	Sensitivity of the group selection	0.644	0.939	0.649	0.98
	Specificity of the group selection	0.684	0.891	0.653	0.989
	Response rate in the sensitive group	0.387	0.469	0.4	0.495
	Run time (min.)	209.2	53.8	310.9	96.15
	Tuning set	B	−	B	−

*Note*: Tuning sets are specified in Appendix A. Abbreviations: CVASD, cross-validated ASD method; CVRS, cross-validated risk scores.

**Table 3:** Operating characteristics of the CVASD and the CVRS methods for different true response rates in sensitive group on the treatment arm (TRR) for the following scenario: 25% response rate on the control arm, 35% response rate in nonsensitive group on the treatment arm, 5% of the patients are sensitive

		Sample size 400		Sample size 1000	
TRR	Operating characteristics	CVASD	CVRS	CVASD	CVRS
60%	Power for overall 0.04 level test	0.609	0.609	0.965	0.965
	Power for sensitive group 0.01 level test	0.161	0.151	0.465	0.429
	Overall power of the design	0.672	0.668	0.981	0.98
	Sensitivity of the group selection	0.737	0.937	0.858	0.996
	Specificity of the group selection	0.754	0.739	0.783	0.946
	Response rate in the sensitive group	0.384	0.41	0.448	0.546
	Run time (min.)	171.5	43.3	264	64.2
	Tuning set	B	−	B	−
50%	Power for overall 0.04 level test	0.552	0.552	0.94	0.94
	Power for sensitive group 0.01 level test	0.116	0.139	0.239	0.314
	Overall power of the design	0.604	0.614	0.954	0.959
	Sensitivity of the group selection	0.581	0.831	0.584	0.962
	Specificity of the group selection	0.786	0.667	0.811	0.83
	Response rate in the sensitive group	0.337	0.381	0.373	0.426
	Run time (min.)	168.1	43.1	258.2	65.1
	Tuning set	B	−	B	−

*Note:* Tuning sets are specified in Appendix A. Abbreviations: CVASD, cross-validated ASD method; CVRS, cross-validated risk scores.

**Table 4:** Operating characteristics of the CVASD and the CVRS methods for different true response rates in sensitive group on the treatment arm (TRR) for the following scenario: 35% response rate on the control arm, 35% response rate in nonsensitive group on the treatment arm, 20% of the patients are sensitive

		Sample size 400		Sample size 1000	
TRR	Operating characteristics	CVASD	CVRS	CVASD	CVRS
60%	Power for overall 0.04 level test	0.129	0.129	0.299	0.299
	Power for sensitive group 0.01 level test	0.138	0.287	0.724	0.8
	Overall power of the design	0.249	0.379	0.807	0.86
	Sensitivity of the group selection	0.838	0.967	0.913	0.993
	Specificity of the group selection	0.812	0.951	0.977	0.999
	Response rate in the sensitive group	0.489	0.572	0.578	0.598
	Run time (min.)	208	66.6.7	303.8	99.1
	Tuning set	C	−	C	−
50%	Power for overall 0.04 level test	0.053	0.053	0.115	0.115
	Power for sensitive group 0.01 level test	0.022	0.071	0.109	0.292
	Overall power of the design	0.074	0.12	0.211	0.373
	Sensitivity of the group selection	0.846	0.886	0.852	0.961
	Specificity of the group selection	0.474	0.812	0.739	0.953
	Response rate in the sensitive group	0.371	0.449	0.42	0.487
	Run time (min.)	204.3	65.4	298.4	99.1
	Tuning set	D	−	D	−

*Note*: Tuning sets are specified in Appendix A. Abbreviations: CVASD, cross-validated ASD method; CVRS, cross-validated risk scores.
