# Supplementary material for: Developing and testing high-efficacy patient subgroups within a clinical trial using risk scores
Source: Stat Med. Author manuscript; Available in PMC 2021 Oct 27. (PMC7611900; doi:10.1002/sim.8665)
Supplement: Supplementary materials [file EMS137080-supplement-Supplementary_materials.pdf]

Supplementary Material for “Developing and testing high-efficacy patient subgroups within a clinical trial using risk scores” by Svetlana Cherlin and James M. S. Wason

Supplementary Table 1 Operating characteristics of the CVASD and the CVRS methods for the scenario with no sensitive group. Response rate in all patients on both arms is 25%. The results are based on 5,000 simulations. The tuning sets for the CVASD are: $(\eta, R, G) = \{(0.8, 1.5, 2), (0.8, 1.6, 1), (0.8, 1.7, 1)\}$.

	**Sample size 400**		**Sample size 1000**	
**Operating characteristics**	**CVASD**	**CVRS**	**CVASD**	**CVRS**
Power for overall 0.04 level test	0.03	0.03	0.035	0.035
Power for sensitive group 0.01 level test	0.061	0.011	0.009	0.015
Overall power of the design	0.089	0.041	0.044	0.05
Sensitivity of the group selection	0.588	0.506	0.245	0.501
Specificity of the group selection	0.59	0.5	0.887	0.5
Response rate in the sensitive group	0.2	0.249	0.211	0.25
Run time (min.)	825	208	1238	315.8

Supplementary Table 2 Operating characteristics of the CVRS design for the data that were simulated with main treatment effect, main covariates effect (prognostic effect) and treatment-covariates interaction effects. The data were simulated assuming that the response rate for the sensitive group under control is 35%, the response rate for the sensitive group under treatment is 70%, and the response rate for the non-sensitive group is 25%. 10 out of 100 covariates are sensitive, 10% out of 400 patients are sensitive. In the analysis, the data were modelled assuming (i) interaction effect only (“T:C" column), (ii) man covariates effect and the interaction effect (“C+T:C" column), (iii) main treatment and covariates effect and the interaction effect (“T+C+T:C" column). The results are based on 1,000 simulations.

**Operating characteristics**	T:C	C+T:C	T+C+T:C
Power for overall 0.04 level test	0.074	0.074	0.074
Power for sens. group 0.01 level test	0.229	0.231	0.210
Overall power of the design	0.284	0.287	0.268
Sensitivity of the group selection	0.995	0.967	0.961
Specificity of the group selection	0.969	0.869	0.860
Response rate in the sensitive group	0.625	0.519	0.506

Supplementary Table 3 Operating characteristics of the CVRS design. The data were simulated assuming a high correlation ($\rho = 0.6$), a low correlation ($\rho = 0.3$) and no correlation ($\rho = 0$) between the covariates. The response rate on the control arm is 25%, the response rate in the non-sensitive patients on treatment arm is 25%, the response rate in the sensitive group on treatment is 70%. 10 out of 100 covariates are sensitive, 10% out of 400 patients are sensitive. The results are based on 1,000 simulations.

**Operating characteristics**	$\rho = 0.6$	$\rho = 0.3$	$\rho = 0$
Power for overall 0.04 level test	0.109	0.122	0.144
Power for sens. group 0.01 level test	0.148	0.225	0.463
Overall power of the design	0.240	0.320	0.540
Sensitivity of the group selection	0.872	0.932	0.996
Specificity of the group selection	0.746	0.834	0.970
Response rate in the sensitive group	0.401	0.468	0.641

Supplementary Table 4 Operating characteristics of the CVASD and the nested CVRS (n.CVRS) designs for different true response rates in sensitive group on the treatment arm (TRR) for the following scenario: 25% response rate on the control arm, 25% response rate in non-sensitive group on the treatment arm, 10% of the patients are sensitive, sample size is 1000. This scenario corresponds to the first simulation scenario presented in the manuscript.

		**Sample size 400**		**Sample size 1000**	
**TRR**	**Operating characteristics**	**CVRS**	**n.CVRS**	**CVRS**	**n.CVRS**
70%	Power for sensitive group 0.01 level test	0.462	0.464	0.976	0.976
	Overall power of the design	0.524	0.526	0.982	0.982
	Sensitivity of the group selection	0.994	0.994	0.998	0.998
	Specificity of the group selection	0.983	0.985	1	1
	Response rate in the sensitive group	0.664	0.668	0.696	0.696
60%	Power for sensitive group 0.01 level test	0.261	0.262	0.828	0.827
	Overall power of the design	0.321	0.323	0.849	0.850
	Sensitivity of the group selection	0.985	0.986	0.996	0.996
	Specificity of the group selection	0.955	0.962	1	1
	Response rate in the sensitive group	0.553	0.562	0.6	0.6
50%	Power for sensitive group 0.01 level test	0.102	0.102	0.451	0.454
	Overall power of the design	0.161	0.162	0.497	0.499
	Sensitivity of the group selection	0.951	0.951	0.991	0.991
	Specificity of the group selection	0.875	0.885	0.988	0.989
	Response rate in the sensitive group	0.428	0.437	0.490	0.491

How to cite this article: Cherlin S., Wason J. M. S. Developing and testing high-efficacy patient subgroups within a clinical trial using risk scores, *Statistics in Medicine*, 2020.

Supplementary Table 5 Covariates with the corresponding treatment-covariate interaction P-values, from the analysis of the START trial. The names of the covariates are coded according to the study of Fonagy et al., 2018.

Covarite	P-value
HLM_NVAP.P1	0.006
HLM_All.Offs.P1	0.007
YP_SRD_PeerDelT1	0.016
YP_SDQ_HyperT1	0.026
P_FACE_FCommT1	0.035
YP_SDQ_ProSocT1	0.042
HLM_OthBr.P1	0.056
IQ	0.079
YP_SDQ_PeerRelT1	0.081
HLM_VAP.P1	0.081
YP_SDQ_EmotT1	0.083
P_ALAB_ParInvT1	0.092
YouthAdolescent	0.116
P_CONN_ADHDTscoreT1	0.128
P_FACE_FSatT1	0.131
YP_ALAB_ParInvT1	0.138

Supplementary Table 6 Number of participants in each arm who are sensitive/non-sensitive for the CVASD and the CVRS methods, from the analysis of the START trial. For each method, the P-value for the interaction effect between the treatment and the sensitivity status is stated. CVRth represents the CVRS method with an additional selection step for the P-value threshold.

	Sensitive group		Non-sensitive group	
**Method**	**Control**	**Treatment**	**Control**	**Treatment**
CVASD, $P = 0.752$	34	36	302	297
CVRS, $P = 0.122$	222	231	114	102
CVRSth, $P = 0.043$	294	290	42	43
